# Sex and Time-of-Day Impact on Anxiety and Passive Avoidance Memory Strategies in Mice

**DOI:** 10.3389/fnbeh.2020.00068

**Published:** 2020-05-25

**Authors:** Ana Belén Meseguer Henarejos, Natalija Popović, Dubravko Bokonjić, Nicanor Morales-Delgado, Antonia Alonso, María Caballero Bleda, Miroljub Popović

**Affiliations:** ^1^Department of Physiotherapy, Faculty of Medicine, University of Murcia, Murcia, Spain; ^2^Department of Human Anatomy and Psychobiology, Faculty of Medicine, University of Murcia, Murcia, Spain; ^3^Institute of Biomedical Research of Murcia (IMIB), Virgen de la Arrixaca University Hospital, University of Murcia, Murcia, Spain; ^4^Medical Faculty of the Military Medical Academy, University of Defense in Belgrade, Belgrade, Serbia; ^5^Department of Histology and Anatomy, Faculty of Medicine, University of Miguel Hernández, Sant Joan Alacant, Spain

**Keywords:** anxiety, mice, passive avoidance memory strategies, sex differences, time-of-day

## Abstract

In humans, anxiety and cognitive processes are age, gender, and time of day dependent. The purpose of the present study was to assess whether the time of day and sex have an influence on anxiety and emotional memory in adult mice. Light-dark and passive avoidance (PA) tests were performed at the beginning and at the end of the light cycle, defined as Zeitgeber time (ZT) ZT0–2.5 and ZT9.5–12, respectively. A baseline difference in anxiety was not found, but on the 24 h retention trial of the PA test, females presented longer latencies to enter into the dark compartment at the ZT0–2.5 time point of the day. The data from the second test day (PA reversal trial) indicated that some animals associated the dark compartment with an aversive stimulus (shock), while others associated the aversive stimulus with crossing from one compartment to another. At the ZT9.5–12, female mice mainly related the aversive stimulus to transferring from one compartment to another, while male mice associated darkness with the aversive stimulus. There was a negative correlation between the frequency of light-dark transitions in the light-dark test and the PA latency on the 24 h retention trial in males tested at ZT0–2.5. The PA latency on the reversal and 24 h retention trials negatively correlated with a risk assessment behavior in male mice tested on ZT0–2.5 and ZT9.5–12, respectively. In conclusion, our data reveal that the impact of motor activity and risk assessment behavior on PA memory formation and applied behavioral strategies are time of day and sex dependent.

## Introduction

The circadian rhythm is a 24-h internal regulator that coordinates physiological and behavioral activities with diurnal environmental variation. Studies based on morningness-eveningness individual preferences indicated the existence of three circadian typologies in humans (morning-, intermediate-, and evening-type; Kerkhof, 1985). Adan and Natale (2002) demonstrated gender differences in circadian typology, indicating that women present a more pronounced morningness preference than men. Moreover, the optimal moment of daily activities for morning-type women appears 2 h earlier (11:00 h) than in morning-type men (13:00 h; Adan and Sánchez-Turet, 2001).

Eveningness is generally negatively related to academic achievement (Preckel et al., 2011; Fabbian et al., 2016) and gender studies have indicated that female secondary school pupils and undergraduate students had better grade point averages (GPA) than male students (Chee et al., 2005; Freudenthaler et al., 2008). Although it is well known that women are about twice more likely to develop generalized anxiety disorders, panic disorders, and posttraumatic stress disorders (Hantsoo and Epperson, 2017), the influence of gender circadian typology on anxiety is not clear. Some data indicated that morningness negatively correlated with anxiety in men but not in women (Matthews, 1988) and vice versa (Díaz-Morales and Sánchez-López, 2008). There also exists inconsistencies in whether anxiety conditions affect academic performance. It has been demonstrated that high anxiety levels could be related to lower academic performance (Seipp, 1991) or higher GPA scores (Rahafar et al., 2016). Furthermore, the timing of examination negatively affected the obtained scores if the exam was performed in a period of the day that did not coincide with individual circadian typology (van der Vinne et al., 2015). This study suggests that exam schedules should be adjusted to the period between 12:45 h and 15:00 h, since this interval gives equal academic opportunities to all chronobiological types.

In mice, the sex differences in anxiety and emotional memory have been a matter of investigation for many years, with unclear conclusions since the results are strain and test specific (O’Leary et al., 2013). In the present article, we will focus on research performed in mice using the light-dark anxiety test and the passive avoidance (PA) memory task. On one hand, the light-dark anxiety test, developed by Crawley and Goodwin (1980), is based on the motivational conflict between exploration of a novel dark area and the natural tendency to avoid a bright-lit space. On the other hand, the PA task, used to measure emotional memory, is based on avoidance of a dark space when it is associated with an adverse event (Ögren and Stiedl, 2015).

Although female C57BL/J6 mice exhibited longer latency than males to enter the dark chamber in the light-dark test (Adamec et al., 2006), most of the studies have proven that there are no sex differences in the time that animals spent in the dark compartment among the following strains of mice: C57BL/6J (Adamec et al., 2006; Huang et al., 2017; Tucker et al., 2017), C57BL/6 (Ding et al., 2014), C57BL/6N (Yokota et al., 2017), NMRI (Salari and Amani, 2017), 1129S2/SvHsd, and C57BL/6JOlaHsd mice (Võikar et al., 2001). However, some other studies have suggested that female C57BL/6J (Carreira et al., 2017) and Swiss–Kunming strain (Guo et al., 2004) mice show higher preferences for the dark compartment.

A similar discrepancy occurs concerning the number of light-dark transitions. There were no sex-differences in this parameter in C57BL/6 (Ding et al., 2014), C57BL/6J-NHsd (Barreto-Estrada et al., 2004), C57BL/6J (Carreira et al., 2017), 1129S2/SvHsd, and C57BL/6JOlaHsd mice (Võikar et al., 2001). Data obtained in FVB/NHsd mice revealed a significantly higher number of light-dark transitions in females than in males (Võikar et al., 2001) and, oppositely, male Swiss mice showed higher levels of crossing and wall rearing than female ones (Venerosi et al., 2010). Testing the risk assessment behavior in the light-dark test, Venerosi et al. (2010) found that female Swiss mice exhibited more risk assessment than males.

With respect to PA learning, no sex differences were found in Swiss (Fernandes-Santos et al., 2012; Jardim et al., 2017), C57BL/6NxBALB/c (Adelöf et al., 2019), C57BL/6J (Benice et al., 2006; Zanos et al., 2015), C57BL/6N (Dachtler et al., 2016), 129 Sv (Maurice et al., 2016), C57BL/6 (Podhorna et al., 2002; Ding et al., 2014), C57BL/6J (B6; Rizk et al., 2005; Balsevich et al., 2014), CD1 (Rayburn et al., 2001; Parra et al., 2013), or NMRI mice (Lamberty and Gower, 1988). Furthermore, Yokota et al. (2017) found that female C57BL/6N mice express a significantly longer latency to enter into the dark chamber of the PA task than their male counterparts.

To our knowledge, the effect of the daytime on PA performance has not been contemplated previously in mice. Even though the aim of several studies in mice was not generally to distinguish between these two variables, it has been found that female ICR mice tested at Zeitgeber time (ZT) ZT2–7 displayed a shorter latency on the retention trial in the step-down PA test than the male ones (Xu et al., 2013), whereas at ZT17–23 there were no sex differences among mice (Xu et al., 2011). The data obtained in Wistar–Imamichi rats indicated better PA performance in the light phase (14:00 h) than in the dark phase (02:00 h) that coincides with a maximum serum corticosterone level at 14:00 h and minimum at 2:00 h (Yamada and Iwasaki, 1994). There were no significant differences in memory performance between rats tested at the beginning and at the end of the light period in the Morris water maze and the context-dependent fear conditioning task (McDonald et al., 2002). However, rats tested at the beginning of the light phase expressed significantly better memory retention in the elevated-plus maze test than rats tested at the end of the light phase (Morales-Delgado et al., 2018).

To establish whether anxiety level can predict performance in emotional memory, Navarro-Francés and Arenas (2014) assigned mice according to their behavior in the elevated plus maze test into three groups: low-, medium-, and high-anxious. They found that there were no sex differences when performing the PA task in animals defined as low- and medium- anxious animals. However, high-anxious females had longer crossing latencies than the male ones. To our knowledge, this study is the first to evaluate both sex and time of day impact on the correlation between anxiety level in the light-dark test and PA behavior.

## Materials and Methods

### Animals

Experiments were carried out on 4-month-old Swiss mice, 36 male (body weight 41.27 ± 2.99 g) and 38 female (body weight 35.63 ± 5.31 g). The mice were housed in large Macrolon cages on sawdust bedding, between four and seven mice per cage (40 × 22 × 18 cm). They were kept in the same air-conditioned room (25 ± 1°C), at 50% humidity and under a 12 h light/12 h dark cycle (light on from 08:00 h to 20:00 h). Food and tap water were available *ad libitum*. One week before the experimental procedure, the mice were handled daily for 5 min each. Bearing in mind that the level of plasma corticosterone in mice is significantly lower at the beginning of the light period than at the end (Kakihana and Moore, 1976), in the present study, the behavioral tests were performed during the following daytime periods: in the morning (08:00–10:30 h; defined as ZT0–2.5) and late afternoon (17:30–20:00 h, defined as ZT9.5–12).

All procedures related to the animal maintenance and experimentation were in accordance with the European Communities Council Directive of November 24, 1986 (86/609/EEC) and the guidelines issued by the Spanish Ministry of Agriculture, Fishing, and Feeding (Royal Decree 1201/2005 of October 21, 2005) and were approved by the Institutional Animal Ethics Committee. All efforts were made to minimize the number of animals used and prevent their suffering. The euthanasia was induced by carbon dioxide inhalation.

### Light-Dark and Passive Avoidance Tests

The experiments were performed in an automatically operated commercial PA Apparatus (Ugo Basile) and video recorded. The PA step-through cage was divided into two equal size compartments: light (white and illuminated by a 24V-10W bulb) and dark (black and dark). The two compartments were separated by a partition which embodies an automatically operated sliding door at the floor level.

With the aim to evaluate the baseline behavior in light-dark conditions, on day one, each mouse was initially placed in the light compartment, facing away from the dark compartment (door open and shock disconnected) and was allowed to explore both chambers for 5 min. During this period, the following behavioral parameters were recorded: *latency to first enter into the dark compartment, latency to first re-enter into the light compartment, time spent in the light compartment, number of the light-dark transitions, and time spent in risk assessment behavior of the light and dark compartment (head and/or incomplete body dipping into either one compartment)*. To facilitate comparisons between groups, the distribution of behavioral activities of risk assessment in the light/dark test was additionally calculated as *“percent of total activity per time spent in the corresponding compartment.”*

Once 5 min time of the light-dark test was completed, when the mouse entered into the dark compartment, the door was closed and a 0.7 mA shock was delivered for 3 s (PA acquisition trial). After 45 s, the mouse was removed from the dark compartment and returned to its home cage. Animals that did not enter into the dark compartment within the first 60 s of light-dark test or expressed less sensitivity to the shock in the PA task (lack of vocalization and/or jumping response), were excluded from the analysis. A total of 29 male (14 animals at the ZT0–2.5 and 15 animals at the ZT 9.5–12) and 36 female mice (18 animals at both ZT0–2.5 and ZT 9.5–12) were included in the statistical analysis.

Twenty-four hours later, the retention and reversal trials were performed. In the retention trial the aim was to avoid the possibility of memory extinction. When the mouse passed from the light into the dark compartment with all four paws in, the automated slide door was closed and a 0.7 mA shock was delivered for 3 s. After 45 s, the mouse was removed from the dark compartment and returned to its home cage for 60 s. The latency to transfer into the dark compartment was measured during a maximum of 540 s (maximum cut off time allowed by the used apparatus). Animals that did not enter into the dark compartment within this period were returned to their cages for 60 s, too. After the retention test, a reversal trial was proceeded, placing the mouse into the dark chamber (door open and shock disconnected) and the latency to transfer into the light compartment was measured with a maximum of 540 s. The cage catch pan, grid floor, and side walls were cleaned with 70% ethanol before each animal was tested and also between trials.

### Statistical Analysis

Descriptive data are presented as median and percentiles (P25, P75). The statistical analysis was performed using SPSS 24.0 software (IBM Corp., Armonk, NY, USA). Since a Shapiro-Wilk test indicated the non-normal distribution of data values, the group differences were analyzed by the Mann-Whitney test. Correlations between parameters assessed in the light-dark and PA tests were analyzed by the Spearman’s correlation coefficient for each group separately (males and females at ZT0.2.5 and ZT9.5–12), followed by the principal component analysis and Varimax rotation. An eigenvalue greater than 1 was set as the criterion for selecting components, whereas factor loadings ≥0.500 are reported. Statistical significance was considered if *p* < 0.05.

## Results

### Light-Dark Test

**Figure 1:**
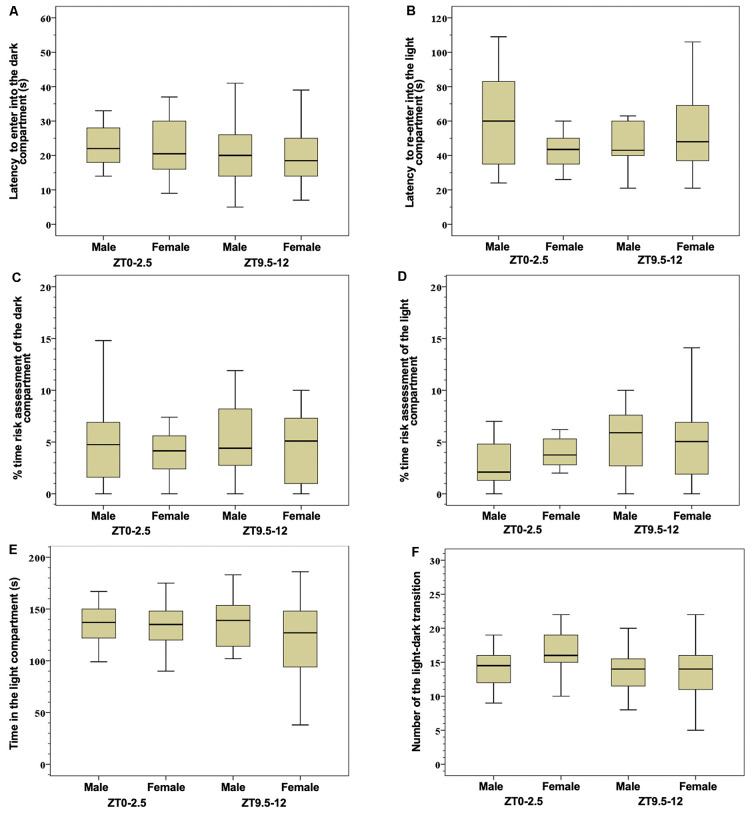
Latency to enter into the dark compartment **(A)** latency to re-enter into the light compartment **(B)**, % time risk assessment of the dark compartment **(C)**, % time risk assessment of the light compartment **(D)**, time spent in the light compartment **(E)**, and number of the light-dark transitions **(F)** in the light-dark test. Box-and-whisker plot showing median (horizontal line inside box), 25 and 75 percentiles (edge of box), and 10 and 90 percentiles (whiskers). There were no significant differences between group in the tested parameters.

There were no significant differences between groups in the parameters tested in the light-dark test (Figure 1). Detailed significant correlations between tested parameters in the light-dark test are summarized in Table 1.

**Table 1:** Spearman’s correlation coefficients (ρ) between behavioral parameters tested in the light-dark and passive avoidance (PA) tasks.

Correlation between	ZT0–2.5						ZT9.5–12					
♂			♀			♂			♀		
	*N* = 14	PAL < 540s *N* = 13	PAL = 540s *N* = 1	*N* = 18	PAL < 540s *N* = 10	PAL = 540s *N* = 8	*N* = 15	PAL < 540s *N* = 10	PAL = 540s *N* = 5	*N* = 18	PAL < 540s *N* = 14	PAL = 540s *N* = 4
Latency enter dark and latency re-enter light	***ρ* = 0.741** *p* **= 0.002**	***ρ* = 0.693** *p* **= 0.009**	-	***ρ* = 0.666** *p* **= 0.003**	***ρ* = 0.844** *p* **= 0.002**					*ρ* = −0.463 *p* = 0.053		
Latency enter dark and risk assessment dark	***ρ* = −0.656** *p* **= 0.011**	***ρ* = −0.641** *p* **= 0.018**	-									
Latency enter dark and risk assessment light	***ρ* = −0.587** *p* **= 0.027**	***ρ* = −0.709** *p* **= 0.007**	-							***ρ* = −0.583** *p* **= 0.011**	***ρ* = −0.577** *p* **= 0.031**	
Latency enter dark and time in light			-	***ρ* = 0.492** *p* **= 0.038**	***ρ* = 0.657** *p* **= 0.039**					***ρ* = 0.620** *p* **= 0.006**	***ρ* = −0.636** *p* **= 0.015**	
Latency enter dark and light-dark transition			-					***ρ* = −0.654** *p* **= 0.040**				
Latency re-enter light and light-dark transition			-				***ρ* = −0.779** *p* **= 0.001**	***ρ* = −0.951** *p* **= 0.001**		*ρ* = −0.466 *p* = 0.051		
Risk assessment light and time in light			-							***ρ* = −0.520** *p* **= 0.027**	***ρ* = −0.570** *p* **= 0.033**	
Latency re-enter light and risk assessment dark	*ρ* = −0.530 *p* = 0.051		-									
Latency re-enter light and risk assessment light	*ρ* = −0.524 *p* = 0.054	***ρ* = −0.664** *p* **= 0.013**	-									
Risk assessment dark and PAL			-				***ρ* = −0.615** *p* **= 0.015**					
Risk assessment light and PAL			-		*ρ* = 0.620 *p* = 0.056		***ρ* = −0.596** *p* **= 0.019**					
Light-dark transition and PAL	***ρ* = −0.646** *p* **= 0.013**	***ρ* = −0.585** *p* **= 0.036**	-									
Latency re-enter light and PA reversal latency			-							***ρ* = −0.730** *p* **= 0.001**	***ρ* = 0.676** *p* **= 0.008**	
Risk assessment light and PA reversal latency		***ρ* = −0.576** *p* **= 0.039**	-				ρ = −0.508 *p* = 0.053					
Risk assessment light and PA reversal latency									*ρ* = −0.872 *p* = 0.054			
Time in light and PA reversal latency			-			***ρ* = −0.898** *p* **= 0.002**						
PAL and PA reversal latency		*ρ* = −0.538 *p* = 0.058	-									

*Only p ≤ 0.060 are presented. Significant correlation is highlighted in bold gray shadow*.

In the group of male mice tested at the ZT0–2.5, there existed a significant negative correlation between the latency to enter into the dark compartment and the risk assessment of both dark and light compartments. In both male and female mice at the ZT0–2.5, a positive correlation between the latency to enter into the dark compartment and latency to re-enter into the light compartment was present.

In male mice tested at the ZT9.5–12, a negative correlation was found between the latency to re-enter into the light compartment and the number of light-dark transitions. Female mice in this tested period showed: (1) a significant positive correlation between latency to enter into the dark compartment and time spent in the light compartment; and (2) a negative correlation between risk assessment of the light compartment and latency to enter into the dark compartment, as well as time spent in the light compartment.

### Passive Avoidance Test

**Figure 2:**
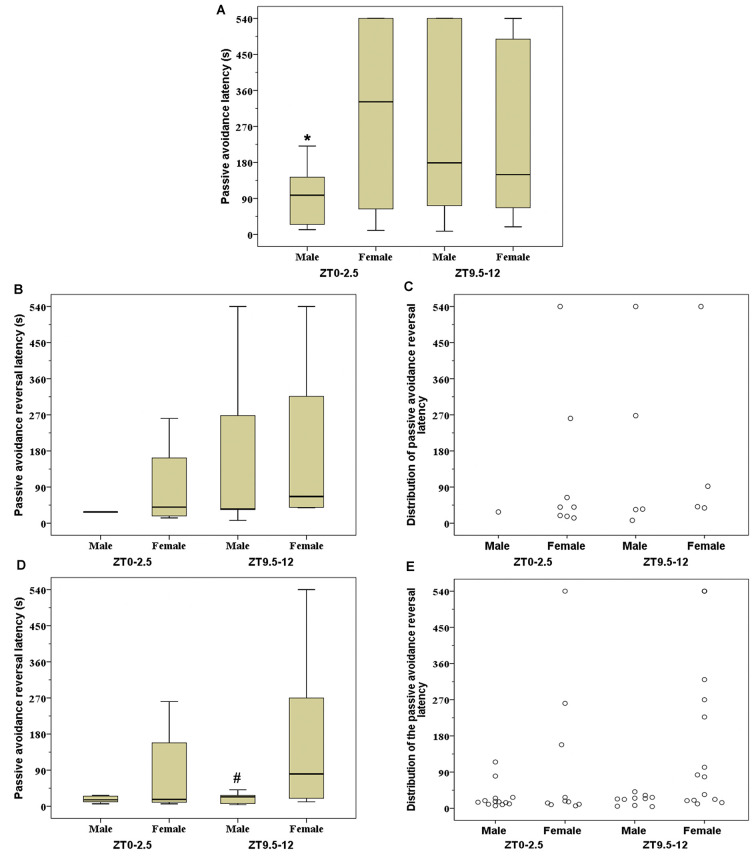
Passive avoidance (PA) latency **(A)**, PA reversal latency **(B)**, and individual distribution of PA reversal latency **(C)** in good performance mice (mice that did not enter into the dark compartment during 540 s on 24 h retention trial); PA reversal latency **(D)** and individual distribution of PA reversal latency **(E)** in mice that entered into dark compartment on 24 h retention trial. Box-and-whisker plot showing median (horizontal line inside box), 25 and 75 percentiles (edge of box) and 10 and 90 percentiles (whiskers). *p < 0.05 vs. female mice at Zeitgeber time (ZT) ZT0–2.5 and ^#^*p* < 0.05 vs. female mice at ZT9.5–12.

The PA latency on the 24 h retention trial was significantly higher in female mice tested at the ZT0–2.5 than in males tested in the same period (*Z* = −2.439; *p* = 0.014; Figure 2A). The latency in the PA reversal trial of animals that entered into the dark compartment on the 24 h retention trial was significantly higher in females than in male mice tested at the ZT9.5–12 (*Z* = −2.021; *p* = 0.043; Figure 2D).

Detailed information of significant correlations between the tested parameters is presented in Table 1. In male mice, there was a significant negative correlation between the PA latency on the 24 h retention trial and three parameters tested in the light-dark test: light-dark transition (ZT0–2.5) and risk assessments of both light and dark compartments (ZT9.5–12). At the ZT0–2.5, in male mice there was a significant negative correlation between latency in the PA reversal trial and the time spent in risk assessment of the light compartment in the light-dark test. During the same period of daylight, the high-performance female rats (animals that did not enter into the dark compartment during retention trial; Figures 2B,C) expressed a significant negative correlation between time spent in the light compartment during the light-dark test and latency to exit from the dark compartment in the PA reversal trial. At ZT9.5–12, a significant positive correlation between the latency to re-enter into light compartment in the light-dark test and latency in the reversal trial was established in the low-performance female mice.

### Principal Component Analysis (Table 2)

For males tested both at ZT0–2.5 and ZT9.5–12, as well as for females tested at the ZT0–2.5, the principal component analysis produced three components that represented 74.55%, 77.08%, and 65.05% of the cumulative variance in the correlation matrix, respectively. However, for females tested at the ZT9.5–12, the principal component analysis produced two components with 59.81% of cumulative variance.

**Table 2:** Principle component analysis with varimax rotation (Kaiser-normalization); criterion for N° of factors extracted: Eigenvalue >1 (Kaiser-Guttman-Criterion); values *x* with −0.100 < *x* < 0.100 are not shown; factor loadings above 0.500 are considered high loading and highlighted in bold gray shadow.

Component Parameter	1				2				3			
	ZT0–2.5		ZT9.5–12		ZT0–2.5		ZT9.5–12		ZT0–2.5		ZT9.5–12	
	♂	♀	♂	♀	♂	♀	♂	♀	♂	♀	♂	♀
Latency to first enter into the dark compartment	**0.571**	0.440		**0.706**	**0.719**	**0.561**	−0.229	**0.531**	−0.251	−0.429	**0.879**	
Latency to first re-enter into the light compartment	**0.780**	0.223		−0.140	0.365	**0.845**	**−0.905**	**0.901**	−0.184	−0.226	−0.296	
Time spent in the light compartment	0.239	**0.738**	−0.136	**0.803**	**0.551**	0.226	**0.816**		**0.595**		0.447	
Number of the light-dark transitions	**−0.685**	0.328	0.155	0.164	−0.111	−0.160	**0.779**	**−0.760**		**0.618**	−0.375	
Time spent in risk assessment behavior of the light compartment		**−0.717**	**−0.773**	**−0.839**	**−0.775**		−0.195	−0.172	−0.222	−0.299	−0.300	
Time spent in risk assessment behavior of the dark compartment	−0.273	−0.129	**−0.796**	**−0.553**	−0.173		0.134	0.224	**0.855**	**0.791**	−0.119	
Passive avoidance latency	**0.904**	−0.162	**0.870**	0.392	−0.150	**0.812**	0.100	−0.253		0.275	−0.184	
Passive avoidance reversal latency		**0.691**	**0.747**	0.223	**0.833**			**0.752**	−0.245	0.114	−0.110	

Component 1 accounted for 29.45% (males at ZT0–2.5), 24.12% (females at ZT0–2.5), 32.50% (males at ZT9.5–12), and 30.04% (females at ZT9.5–12) of the variance. In males tested at ZT0–2.5, this component was mainly loaded by PA latency, latency to first re-enter into the light compartment, the number of light-dark transitions, and latency to first enter into the dark compartment. However, component 1 in females tested at the same period was loaded by time spent in the light compartment, time spent in risk assessment behavior of the light compartment, and PA reversal latency. In males tested at ZT9.5–12, component 1 was loaded by PA latency and PA reversal latency, while in females was loaded by latency to first enter into the dark compartment and time spent in the light compartment. In both sexes, component 1 was loaded by time spent in the risk assessment behavior of the light and dark compartments.

Component 2 accounted for 28.91% (males at ZT0–2.5), 22.18% (females at ZT0–2.5), 27.70% (males at ZT9.5–12), and 29.77% (females at ZT9.5–12) of the variance. In males tested at ZT0–2.5, component 2 was loaded by time spent in the light compartment, time spent in risk assessment behavior of the light compartment, and PA reversal latency. In females tested at the same daytime period, component 2 was loaded by latency to first re-enter into the light compartment and PA latency. In both males and females at ZT0–2.5, component 2 was loaded by the latency to first enter into the dark compartment. In males tested at ZT9.5–12, component 2 was loaded by time spent in the light compartment while in females it was loaded by the latency to first enter into the dark compartment and PA reversal latency. In both sexes, component 2 was loaded by the latency to first re-enter into the light compartment and the number of light-dark transitions.

Component 3 accounted for 16.19% (males at ZT0–2.5), 17.76% (females at ZT0–2.5), and 16.88% (males at ZT9.5–12) of the variance. Component 3, in males tested at ZT0–2.5, was loaded by time spent in the light compartment, while in females was loaded by the number of light-dark transitions. In both sexes, component 3 was loaded by time spent in the risk assessment behavior of the dark compartment. Component 3, in males tested at ZT9.5–12, was loaded by the latency to first enter into the dark compartment.

## Discussion

The results of the present study reveal that there are no significant sex differences in mice tested in the light-dark test at the beginning and at the end of the light phase (test period: ZT0–2.5 and ZT9.5–12) of the 12/12cycle. Similarly, other studies performed at some points of the beginning-middle [ZT2-ZT5 (Võikar et al., 2001) and ZT3.5–6.5 (Huang et al., 2017)] or middle-end period of the light phase [ZT5–10 (Salari and Amani, 2017)] revealed no significant differences between male and female mice in parameters tested in the light-dark test. These findings were also confirmed by some other studies that did not specify the exact period of the light phase when the experiments were performed (Ding et al., 2014; Tucker et al., 2017; Yokota et al., 2017). During the dark phase, Barreto-Estrada et al. (2004) demonstrated that there were no sex differences in the light-dark transition. However, another study performed at ZT14–20 of the dark phase found higher levels of crossing and wall rearing in males than in females and, oppositely, female mice expressed more risk assessment than male ones in the light-dark test (Venerosi et al., 2010).

Further, the present data show that most of the correlations are sex dependent, confirming the results obtained by Carreira et al. (2017). However, we need to point out that at the ZT0–2.5, in both male and female mice, there existed a positive correlation between the latency to enter into the dark compartment and the latency to re-enter into the light compartment. It may, therefore, indicate that at the beginning of the light phase of the light-dark cycle, the motivation for exploration is similar in both sexes. Moreover, in both sexes, correlations between parameters in the light-dark test at ZT0–2.5 do not coincide with correlations at ZT9.5–12. Factor analysis performed by Carreira et al. (2017) indicated that light-dark transition in males could be considered as locomotion while in females could not be considered as part of either anxiety or locomotion. The factor analysis presented herewith indicates that one parameter can relate to several components depending on the sex and testing period, suggesting that it can reflect different types of behavior. Occasionally, there are parameters loading the same component in both testing periods in each sex. Thus, at both ZT-testing periods, the time spent in the light compartment (considered as anxiety-like behavior) loads to component 1 in females and component 2 in males. Furthermore, the time spent in risk assessment behavior of the light compartment (usually considered as anxiety-like behavior) loads to component 1 in females, while in the same animals, the latency to first enter into the dark compartment and the latency to the first re-enter into the light compartment, loads to component 2. Altogether, the present data suggest that even when there are no main sex and time of day effects on anxiety-like, motor and explorative behavior, the correlations between parameters as well as components loading are sex and daytime specific.

In addition to the above, most of the studies have suggested that there are no sex differences in PA memory in mice tested during the light (Rayburn et al., 2001; Rizk et al., 2005; Benice et al., 2006; Fernandes-Santos et al., 2012; Balsevich et al., 2014; Zanos et al., 2015; Dachtler et al., 2016; Maurice et al., 2016; Jardim et al., 2017; Adelöf et al., 2019) and dark phase of the 12/12 cycle (Podhorna et al., 2002; Xu et al., 2011; Parra et al., 2013). In contrast, Xu et al. (2013) found that female ICR mice tested at ZT2–7 showed shorter latency than males on the retention trial in the step-down PA test, while Yokota et al. (2017) found that female C57BL/6N mice displayed significantly longer latency to enter into the dark chamber of the step-through PA task than the male counterparts. The data from the present study showed that female mice performed significantly better than males at the ZT0–2.5 while at the ZT9.5–12 there were no sex differences in the PA latency. Yokota et al. (2017) also demonstrated that the higher motor activity of males, in front of females, could be beneficial for males in the performance of the active avoidance test, but at the same time increases males’ exposure to threat in the PA test and, consequently, the purest performance in this test. In this sense, it should be kept in mind that at the ZT0–2.5 period a significant negative correlation between the PA latency and the light-dark transition in males was present; the mobility in the threat conditions may be one of the reasons for poor male performance. In the same line, Yokota et al. (2017) found that females in proestrus and estrus express significantly longer latencies than males.

Even though it is generally accepted that the PA task is based on the avoidance of the dark space associated with an adverse event (Ögren and Stiedl, 2015), Randall and Riccio (1969) suggested that animals may learn the same response in the PA task on completely different bases, using one of three potential approaches: (1) animal is punished for making the particular response (i.e., relates light-dark crossing with punishment); (2) animal is punished for moving; and (3) animal learning is based on classical fear conditioning to distinct situational cues (i.e., relates dark compartment with punishment). The present data (Figures 2C,E), obtained on the PA reversal trial, suggest that females (ZT9.5–12) show a significant tendency to relate the light-dark crossing with an adverse experience. In contrast, male mice, in both testing periods, apparently relate the dark compartment with punishment. Further, the present data suggest that in the period when there are no sex differences in a used PA strategy, the female mice performed better in the 24 h retention trial than their male counterparts, while when the strategy is different, there are no differences in latency outcome at the 24 h retention trial.

Navarro-Francés and Arenas (2014) demonstrated that high-anxious females had longer crossing latencies than male ones. In the present study, the elevated anxiety level in the light-dark test increased the poor performance in the PA test in male mice at the ZT9.5–12 and contributed to the association of the dark compartment with punishment at ZT0–2.5.

In summary, the oscillation of memory formation over the course of day across taxa has been demonstrated on molecular, anatomical, physiological, and behavioral levels (Gerstner and Yin, 2010; Smarr et al., 2014). The effect of the stressful situation on memory formation may vary depending on the stress intensity and the period of the day (Santori et al., 2019). Furthermore, emotional memory formation in fear conditioning is more prone during the inactive phase (Albrecht and Stork, 2017). To our knowledge, this is the first study to show that sex differences in the correlation between anxiety and emotional memory in mice tested in the light-dark and PA tests may be conditioned by the daytime period. We also suggest that individual and sex differences in the applied strategies in resolving memory tasks should be taken into account. This fact reflects the potential relevance for sex differences in the pathogenesis and therapy of neuropsychiatric disorders. Thus, further studies will be required to determine whether the time of the day and the phases of the estrous cycle may influence PA retention strategies.

## Data Availability Statement

The raw data supporting the conclusions of this article will be made available by the authors, without undue reservation.

## Ethics Statement

The animal study was reviewed and approved and all procedures related to the animal maintenance and experimentation were in accordance with the European Communities Council Directive of November 24, 1986 (86/609/EEC) and the guidelines issued by the Spanish Ministry of Agriculture, Fishing and Feeding (Royal Decree 1201/2005 of October 21, 2005) and were approved by the Institutional Animal Ethics Committee (University of Murcia). All efforts were made to minimize the number of animals used and prevent their suffering.

## Author Contributions

AM and MP performed the experiments. NP, MC, and MP analyzed the video records. DB, NM-D, AA, and MP managed the literature searches. DB and MP undertook the statistical analysis and data presentations. AM, NP, DB, NM-D, AA, MC, and MP contributed to drafting the work and have approved the final manuscript.

## Conflict of Interest

The authors declare that the research was conducted in the absence of any commercial or financial relationships that could be construed as a potential conflict of interest.
